# Liver Damage and Exposure to Toxic Concentrations of Endogenous Retinoids in the Pathogenesis of COVID-19 Disease: Hypothesis

**DOI:** 10.1089/vim.2020.0330

**Published:** 2021-08-13

**Authors:** Anthony R. Mawson, Ashley M. Croft, Federico Gonzalez-Fernandez

**Affiliations:** ^1^ Department of Epidemiology and Biostatistics, College of Health Sciences, Jackson State University, Jackson, Mississippi, USA.; ^2^ School of Pharmacy and Biomedical Sciences, University of Portsmouth, England, United Kingdom.; ^3^ Department of Ophthalmology and Pathology, University of Mississippi Medical Center, Jackson, Mississippi, USA.; ^4^ Department of Research & Development, GV (Sonny) Montgomery Veterans Affairs Medical Center, Jackson, Mississippi, USA.

**Keywords:** SARS-CoV-2, COVID-19, pathogenesis, liver, pathology, retinoids, vitamin A, metabolic syndrome

## Abstract

Severe acute respiratory syndrome coronavirus 2 (SARS-CoV-2) has a marked tropism for the biliary tract; it damages the bile ducts and hepatocytes and can lead to liver decompensation, cirrhosis, and sepsis. The pathogenesis of liver damage and its association with damage to the lung, heart, and brain and to the other protean manifestations of COVID-19 disease are not fully understood. In particular, tissue damage from thinning and leaky blood vessels appears to result from an inflammatory response to the virus rather than the virus itself. This article outlines a new hypothesis of the nature of the inflammatory factor responsible for tissue damage in COVID-19. Review of the literature reveals that COVID-19 disease closely resembles an endogenous form of hypervitaminosis A. We propose that SARS-CoV-2 virus-induced liver damage causes retinoic acid and stored retinyl esters to be released into the circulation in toxic concentrations, unbound to protein, with resulting damage to organs including the lungs, heart, blood vessels, and skin. Several lines of evidence support this model of disease causation. Subject to testing, strategies for the effective treatment and prevention of COVID-19 could include targeting the action and accumulation of retinoids.

## Background

Severe acute respiratory syndrome coronavirus 2 (SARS-CoV-2), the virus that causes COVID-19 disease, has a marked tropism for the biliary tract and is associated with sinusoidal congestion, cell-mediated apoptotic hepatitis, and damage to bile ducts and hepatocytes. In severe cases this is followed by decompensation, cirrhosis, and sepsis (30,37). Postmortem liver biopsy specimens of patients with COVID-19 have also shown moderate microvesicular steatosis and mild lobular and portal activity, suggesting a direct cytotoxic effect of the virus on the liver (38). The precise mechanisms of the pathogenesis and how liver injury relates to distant organ damage and the other manifestations of COVID-19 disease remain unknown. Tissue damage from thinning and leaky blood vessels appears to result from an inflammatory response to the virus rather than from the virus itself. For instance, in a study of the brains of 19 patients who died shortly after contracting COVID-19, there was evidence of microvascular blood vessel damage but no evidence of SARS-CoV-2, suggesting that the neurological damage was caused by a disease-induced inflammatory response rather than by a direct viral attack on the brain (17). This article presents empirical evidence suggesting that the inflammatory factor responsible for tissue damage in COVID-19 is a high concentration of retinoic acid or retinyl esters.

Clues to the pathogenesis that we propose include the fact that >80% of vitamin A is stored in the stellate cells of the liver and its accumulation can lead to their activation and hypertrophy, followed by fibrosis and liver injury. Under normal circumstances vitamin A mobilization occurs through retinyl ester hydrolysis and its secretion into the circulation as retinol-binding protein 4 (RBP4). The latter delivers its retinol cargo by binding to surface receptors (STRA6) at the target tissue. The liver release and targeting of retinol is under tight homeostatic control, so that even under conditions of hypervitaminosis A serum retinol levels may be normal or low, on account of feedback inhibition (25,33). By contrast to the normally highly regulated secretion and targeting of retinol, retinoid mobilization and release are uncontrolled in HBV infection (22). Severe retinoid toxicity can result from sudden shifts in vitamin A stores and its distribution and release into the circulation unbound to protein. The retinoids principally implicated in causing toxicity are retinoic acid and retinyl esters (as retinyl palmitate and stearate) (33). There is evidence that viral hepatitis can be associated with acute hypervitaminosis A, indicated by high serum and liver concentrations of vitamin A but decreased serum RBP4 (13,20).

Building on these observations, we propose that infection-induced activation of the retinoid cascade triggers cell-mediated apoptotic hepatitis, leading to transient cholestatic liver dysfunction, in which stored vitamin A compounds (retinyl esters and the metabolite retinoic acid) enter the circulation through damaged bile ducts and hepatocytes; this exposure can in turn cause lung injury and other organ damage by apoptosis, necrosis, and acute neutrophilic infiltration (21). On this hypothesis, an endogenous form of retinoid toxicity contributes to the multiple signs and symptoms of COVID-19, and disease severity is directly proportional to the concentration of circulating retinyl esters and retinoic acid.

Additional evidence for the hypothesis is presented under six broad categories as follows:

### Syndromic equivalence

There are striking parallels between the features of COVID-19 and those of hypervitaminosis A; these range from flu-like symptoms (fever, cough, muscle aches, fatigue, dyspnea, headache, sore throat, nausea, and vomiting) (4), to androgenetic alopecia (8), bone pain (31), angular cheilitis, erythema (10), altered taste and smell (16), and Guillain–Barré syndrome (9,23,28). The universal clinical observation that COVID-19 involves multiple organ systems suggests a generalized toxic effect. This effect is hypothesized to represent an endogenous state of hypervitaminosis A (21).

### Epidemiological characteristics

On this hypothesis the markedly increased susceptibility of the aged and of patients with metabolic syndrome disorders to COVID-19 is owing to increased concentrations of vitamin A in the liver in these high-risk groups. Preexisting high reserves of vitamin A may increase the substrate for SARS-CoV-2-induced interaction with vitamin A-containing cells, causing their activation and hypertrophy and increasing the overall risk of injury from hypervitaminosis A. Aging is associated with increased vitamin A accumulation in the liver, as shown by increased postprandial plasma retinyl ester concentrations owing to delayed plasma clearance of retinyl esters in triglyceride-rich lipoproteins (15).

Conversely, the strikingly low susceptibility of young children to SARS-CoV-2 infection and to severe COVID-19 (18) may be owing to their naturally low hepatic reserves of vitamin A. Liver samples taken at autopsy from 170 American children 0–15 years of age who died from various causes showed that the median liver vitamin A concentration at birth was low (11 *μ*g retinol/g), remained constant to 3 months, increased rapidly to age 4 years (130 *μ*g/g), and then remained unchanged into adolescence (24).

### Histological evidence

Nonalcoholic fatty liver disease (NAFLD), considered the hepatic manifestation of the metabolic syndrome, plays a key role in insulin resistance and diabetes (11). Expansion of active fat during metabolic abnormalities is paralleled by inflammatory changes, insulin resistance, and fat accumulation in the liver (26). Regarding the link between metabolic disorders and COVID-19 (5), poorer outcomes in COVID-19-infected patients with NAFLD and related metabolic abnormalities may be owing to increased hepatic concentrations of vitamin A in these patients. This hypothesis is supported by the observation that a genetic variant of patatin-like phospholipase domain-containing protein 3 (PNPLA3) is the most prominent heritable factor associated with low serum retinol but with enhanced retinyl esters in the liver of patients with NAFLD (14). This suggests that low serum retinol concentrations typically reported in NAFLD have been misinterpreted as indicating vitamin A deficiency (29); they may instead reflect hypervitaminosis A and impaired hepatic mobilization and secretion. Of interest, this PNPLA3 I148M variant is associated with more severe diseases on the spectrum of NAFLD patients, including subclinical atherosclerosis (19).

### Cirrhosis as a feature of COVID-19

Retinoid toxicity mediated through retinyl esters and/or through retinoic acid, the major metabolite of vitamin A, may also explain the initiation and worsening of existing liver decompensation in severe cases of COVID-19. Cirrhosis and multiorgan failure, commonly seen in COVID-19, may be secondary to an endogenous form of hypervitaminosis A. This is supported by a study of vitamin A profiles in patients with cirrhosis (36). Median retinol levels in the cases were significantly lower than those of the controls (166 *μ*g/L vs. 259 *μ*g/L) but serum retinyl esters were significantly higher (42 *μ*g/L vs. 18 *μ*g/L, *p* < 0.001). An accepted indicator of vitamin A toxicity is serum retinyl esters as a fraction of total vitamin (retinol plus esters) >10% (34). It is noteworthy that the percentage of retinyl esters to total vitamin A in this study (i.e., 42 *μ*g/L/[166 + 42] × 100%) was 20%, that is, double the threshold level of vitamin A toxicity.

### Corticosteroids in COVID-19

Three multicenter randomized controlled trials, along with a meta-analysis, have shown that corticosteroid therapy is of benefit for critically ill patients with COVID-19, regardless of whether they are receiving concurrent mechanical ventilation or oxygen by mask and without mechanical ventilation (27). It is known that corticosteroids inhibit retinoic acid (1). A published case report showing the inhibitory effect of corticosteroids on retinoic acid includes both a source of exposure to retinoids and to one of the hallmarks of severe COVID-19 disease:

A 62-year-old woman with acute promyelocytic leukemia was treated with all-trans retinoic acid. On day 2 she suffered with dyspnea and general fatigue. Marked hypoxia suggested the occurrence of retinoic acid syndrome. She underwent endotracheal intubation and mechanical ventilation with the administration of dexamethasone. Her symptoms promptly abated. She was subsequently treated with conventional chemotherapy and achieved complete remission (6).

These data support our hypothesis that corticosteroids are effective in the treatment of COVID-19 by reducing or inhibiting retinoic acid.

### COVID-19 and interferon

A distinct clinical entity consisting of a highly impaired interferon (IFN) type I response has been observed in critically ill patients with COVID-19, characterized by the absence of IFN-*β* and by low IFN-*α* production associated with a persistent blood viral load and inflammatory response (12). Additional recent evidence indicates that ∼14% of cases of severe COVID-19 either have genetic flaws in IFN production or antibodies that attack IFN itself, leading to reduced serum IFN or an inability to produce IFN (2,39). Although retinoic acid can potentiate the action of IFN on viral replication (32), supplementary or excess retinoic acid can have the opposite effect of impairing immune function and exerting toxic effects regarding oxidative metabolism and mitochondrial function (7).

Observations of impaired IFN production in COVID-19 are consistent with our model of SARS-CoV-2-induced liver damage and resulting retinoid toxicity, because it has been known for many years that retinoic acid can inhibit IFN in a dose-response manner (3). Our hypothesis suggests that genetic flaws in IFN production may enhance retinoid toxicity and hence the severity of COVID-19 disease, and that impaired IFN production may be part of the intermediate mechanism linking retinoid toxicity to poor outcomes associated with SARS-CoV-2 infection.

## Testing the Retinoid Toxicity Hypothesis

**FIG. 1.**
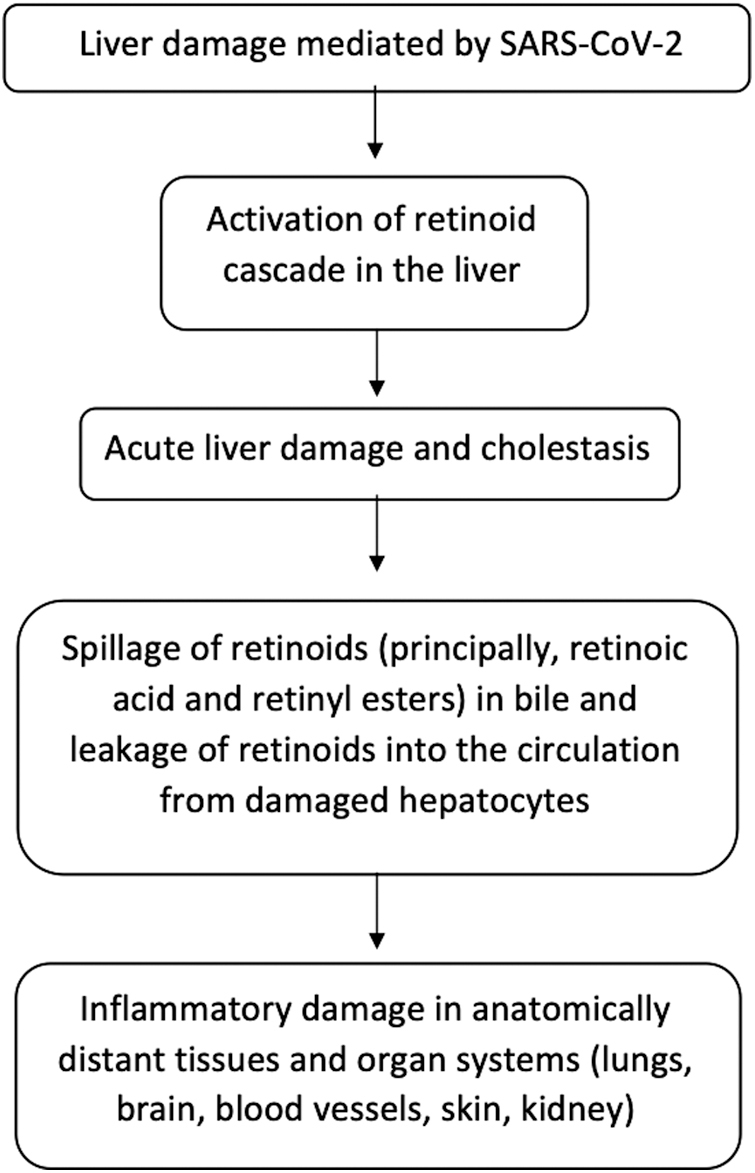
Proposed model of Covid-19 pathogenesis. SARS-CoV-2, severe acute respiratory syndrome coronavirus 2.

In summary, our hypothesis is that COVID-19 is primarily a disease of the liver and biliary system. Its pathogenesis (Fig. 1) arises in liver damage mediated by SARS-CoV-2 and resulting in overspill from the liver and biliary system and into the general circulation of endogenous retinoids (principally, retinoic acid and retinyl esters) in toxic concentrations. Through hematogenous carriage, these toxic molecules cause inflammatory damage in the lungs, brain, blood vessels, skin, and other anatomically distant tissues and organ systems. COVID-19 is likely to be more severe in people with preexisting liver damage from drugs (including alcohol) or from chronic disease, or both, and in those who have specific genetic susceptibilities (35).

Our hypothesis could be readily tested by comparing the retinoid profiles (of serum retinol, retinyl esters, and retinoic acid) in confirmed COVID-19 cases with those of age-matched controls. Cases would be expected to have normal or low concentrations of retinol, associated with liver dysfunction, along with high concentrations of retinyl esters as a percentage of total vitamin A, and increased concentrations of retinoic acid.

If confirmed, our model of disease pathogenesis has important implications for COVID-19 prevention and for its clinical management. Subject to experimental confirmation of their efficacy and safety, treatment strategies would focus on reducing circulating retinoid concentrations (by, e.g., discontinuing all nonessential drugs that are metabolized in the liver and also avoiding all liver-damaging medications in the acute phase of treatment), and on increasing IFN activity.
